# Three-dimensional corrective exercise therapy for idiopathic scoliosis: study protocol for a prospective non-randomized trial

**DOI:** 10.1186/s12891-022-05057-7

**Published:** 2022-02-05

**Authors:** Xuan Zhou, Xin Li, Qikai Wu, Juping Liang, Haibin Guo, Mengdie Jin, Xiaoqing Zhu, Qing Du

**Affiliations:** 1 Department of Rehabilitation, Xinhua Hospital, School of Medicine, Shanghai Jiaotong University, 1665 Kongjiang Road, Shanghai, 200092 China; 2 School of Kinesiology, Shanghai University of Sport, Shanghai, 200438 People’s Republic of China; 3 Xinhua Hospital, Shanghai Jiao Tong University School of Medicine, Shanghai, 200092 People’s Republic of China; 4 Department of Rehabilitation, Chongming Branch of Xinhua Hospital, School of Medicine, Shanghai Jiaotong University, Shanghai, 202150 China

**Keywords:** Idiopathic scoliosis, Three-dimensional corrective exercise, Cobb angle, Physical conditions, Quality of life

## Abstract

**Background:**

Although physiotherapeutic scoliosis-specific exercises (PSSEs) with or without other conservative treatments may improve scoliotic deformities, the evidence is insufficient. Three-dimensional corrective exercises (TDCEs) for scoliosis are based on the theory of PSSEs and are characterized by a combination of outpatient treatment and home-based exercise. This study aims to evaluate the effectiveness of TDCEs for idiopathic scoliosis (IS).

**Methods:**

The participants will be divided into two age- and sex-matched groups: an experimental group (EG) treated with three-dimensional corrective exercise therapy (TDCET) and a control group (CG) receiving generalized exercise therapy. In each arm, mild and moderate IS cases will be reclassified based on the Cobb angle and biopsy results. The primary endpoint is the change in the largest Cobb angle; the secondary endpoints are the sagittal index, forced vital capacity, peak oxygen uptake, and peak oxygen uptake. Sixty-six patients with mild IS and 78 patients with moderate IS will be included.

**Discussion:**

This study is the first controlled trial to systematically assess the effectiveness of TDCEs for IS. In addition to TDCET including three-dimensional corrective exercises, family rehabilitation and basic body awareness therapy may help patients adopt supportive attitudes and appropriate behaviours, thus enhancing their compliance with exercises and achieving better outcomes.

**Trial registration:**

The study protocol was registered at www.clinicaltrials.gov (number identifier: NCT04539522). Registered on August 27, 2020.

**Supplementary Information:**

The online version contains supplementary material available at 10.1186/s12891-022-05057-7.

## Background

Scoliosis is defined by a lateral curvature of the spine (Cobb angle) of at least 10 degrees observed through anteroposterior direct radiography, and this curvature is typically associated with trunk rotation [1]. Idiopathic scoliosis (IS) is the most common clinical entity with an unclear aetiology, and overall prevalence has been reported to be 0.47–5.2% in the current literature [2]. Almost 10% of individuals with adolescent idiopathic scoliosis (AIS) require some form of treatment, and as many as 0.1% curvatures progress to a Cobb angle exceeding 45–50° may eventually require surgery [3]. In addition to the physical implications of IS such as postural changes, an altered appearance, pain, respiratory symptoms and cardiopulmonary dysfunction [4–6], research has shown that this patient group can experience other issues that affect quality of life and lead to psychosocial difficulties [1], including restricted social participation with increased psychiatric consultations, and these patients have been associated with higher rates of eating disorders and suicide [7].

A spinal curvature exceeding 30° is considered a risk factor for health disorders in adulthood [8]; therefore, scoliosis-related scientific societies (i.e., the Scoliosis Research Society [SRS] and the International Society on Scoliosis Orthopaedic and Rehabilitation Treatment [SOSORT]) set a 30° Cobb angle as the best achievable goal for conservative treatments, which includes observation, physiotherapeutic scoliosis-specific exercises (PSSEs), and bracing [9].

Some experts propose PSSEs as compensatory strategies with bracing to prevent potential adverse effects on the muscles and spine as well as to increase brace effectiveness. Moreover, PSSEs can be used as a stand-alone treatment with milder curves to avoid bracing. The latest review concluded that insufficient evidence is available to suggest that PSSEs with or without other conservative treatments can reduce the Cobb angle and improve trunk appearance and balance, [10] and three other reviews of studies conducted between 2005 and 2020 suggested that the few clinical trials on the effects of PSSEs on scoliotic deformities that exist are of low quality [11–13]. Surprisingly, Cantele et al. affirmed that quality of life and self-perception can be impaired in scoliotic girls, especially when they practise PSSEs, because PSSEs are often perceived by adolescents as stressful, boring and limiting to other everyday activities, even when specific exercise programmes may help prevent scoliosis curve progression and improve their health and fitness status when routinely performed [14].

Three-dimensional corrective exercises (TDCEs) for scoliosis are based on the theory of PSSEs and are characterized by the combination of outpatient treatment and home-based exercise: 1) the outpatient treatment component includes three-dimensional self-correction, stabilization of the corrected posture, stretching, manual fascia relaxation therapy, and breathing training, and 2) the home-based exercise component includes active self-correcting exercises combined with basic body awareness therapy. However, according to data from previous randomized controlled trials (RCTs) regarding exercise or bracing, RCTs are not very feasible because of ethical reasons and the high rate of families refused randomization and stated preference for one treatment over the other [15, 16]. Ferriter et al. reported that a non-randomized controlled trial (non-RCT) of high quality can produce outcomes that approximate those found in RCTs [17]. Therefore, a non-RCT was designed to test the following hypothesis: TDCEs are effective in patients with either mild or moderate IS.

**Fig. 1:**
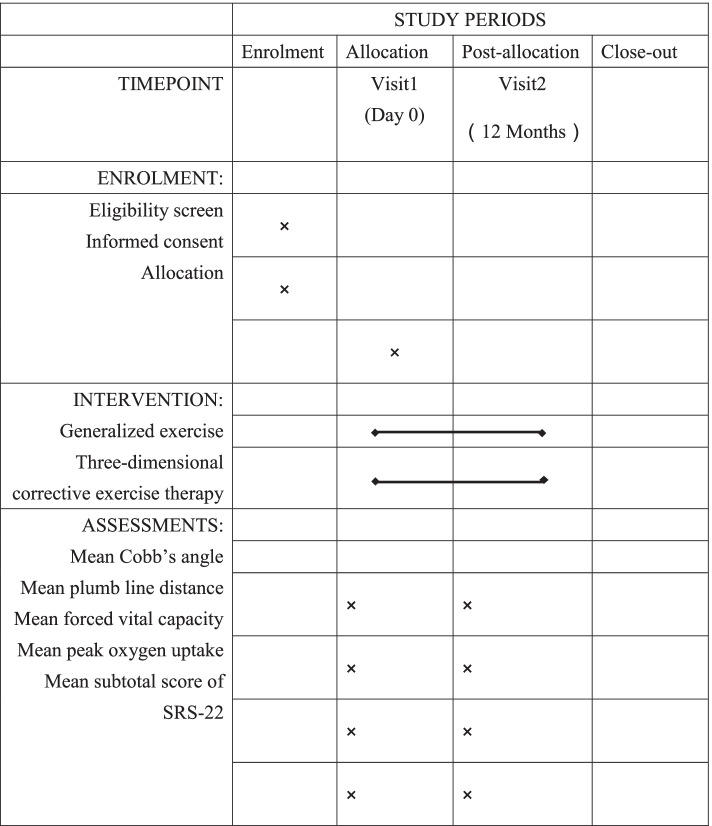
SPIRIT figure

This quasi-experimental prospective longitudinal trial aims to evaluate the effectiveness of a 6-month TDCEs programme versus a control programme for IS. Physical conditions and quality of life will be measured before and after the 12-month intervention to quantify changes and treatment effects. Furthermore, the rehabilitation process will be assessed carefully regarding goal setting and attainment. The flow diagram is shown in Fig. 1.

## Methods/design

Approval and registration of the study is confirmed. The design of this clinical trial follows the recommendations of the SPIRIT guidelines (2013). The conditions described lay a solid foundation for the feasibility of this trial. The trial was registered prospectively at www.clinicaltrials.gov (NCT04539522).

### Participants and setting

We will recruit 144 Patients from the outpatient clinic of the Rehabilitation Department in Xinhua Hospital in the Shanghai Jiao Tong University School of Medicine, Shanghai, China. The department is one of the first scoliosis rehabilitation centres in Shanghai, and the clinicians have a great deal of experience treating IS using conventional methods, especially with regard to exercise therapy. Annually, approximately 1000 IS outpatients visit the department, and approximately 200 of them receive outpatient treatment 1–3 times a week. In addition, the paediatric outpatient department is open for 12 h per day, which is convenient for patients. The conditions described lay a solid foundation for the feasibility of this trial.

### Participant recruitment

Both mild and moderate IS patients from the outpatient clinic will be recruited and stratified by disease severity. Experienced clinicians will divide the participates who meet the inclusion criteria into mild or moderate subgroups according to Cobb angle on the anteroposterior and lateral whole-spine X-rays in the standing position taken at the first visit. Written informed consent will be obtained from participants who meet all the inclusion and none of the exclusion criteria and the parents. Then, participants will be assigned to two age- and sex-matched groups. No further recruitment measures will be taken, and no individuals with a direct relationship with the researchers, such as students/staff of the hospital or close relatives, will be included. Patient enrolment started in November 2020 and is scheduled to end in December 2021.

### Eligibility criteria

Participants must meet all of the following criteria for inclusion:

1. IS with a Cobb angle greater than or equal to 10 degrees and less than 45 degrees
2. Aged between 8 and 16 years old

Cobb angles of less than and greater than 25 degrees on the anteroposterior and lateral whole-spine X-rays taken in the standing position are classified as mild and moderate scoliosis, respectively, according to the SOSORT guidelines.

- Mild IS is defined as a Cobb angle of not less than 10 degrees but less than 25 degrees
- Moderate IS is defined as a Cobb angle of not lower than 25 degrees but less than 45 degrees

### Exclusion criteria

Participants will be excluded if they meet one of the following criteria:

1. An age < 8 years or > 16 years
2. Scoliosis caused by congenital, postural, neuromuscular or other diseases (such as neurofibromatosis, Marfan syndrome, bone dysplasia, metabolic or endocrine diseases, etc.)
3. The apical vertebrae being located at T7 or above
4. A history of pulmonary, cardiovascular, myo-articular, or neurological diseases
5. A history of cognitive and/or physical impairments that would preclude the patient from performing either the exercise therapy or the tests for the study’s outcome measures
6. A history of rehabilitation or surgery before the first visit
7. Complete ossification of the entire growth plate

### Participant timeline

We adhered to the Standard Protocol: Recommendations for Interventional Testing (SPIRIT) in the development and reporting of our protocol (Fig. 1) [18].

### Recruitment procedure

**Fig. 2:**
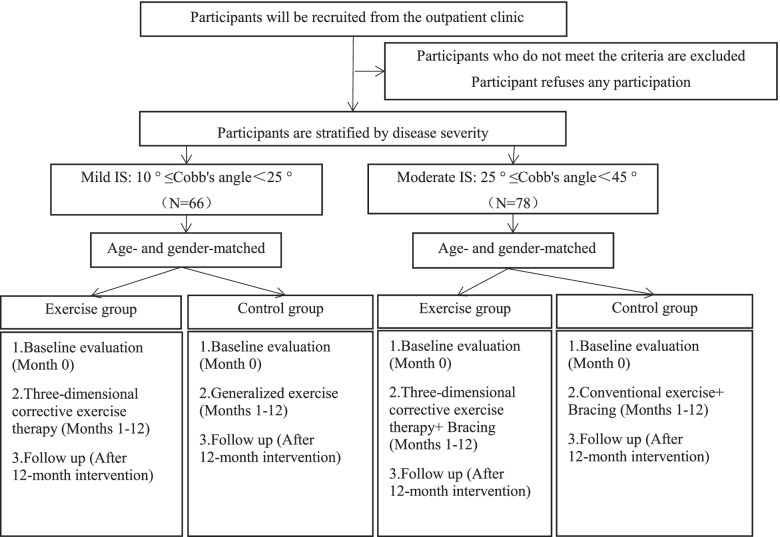
Timeline of the study

The participants will be divided into two age- and sex-matched groups: an experimental group (EG) treated with three-dimensional corrective exercise therapy (TDCET) and a control group (CG) receiving generalized exercise therapy. In each arm, mild and moderate IS cases will be reclassified based on the Cobb angle and biopsy results. Eligible patients will be treated and observed for a total of 12 months (Fig. 2).

#### Three-dimensional corrective exercise therapy

**Fig. 3:**
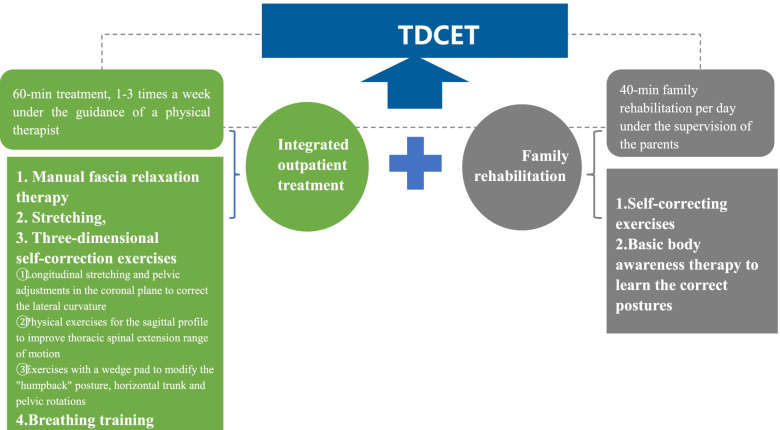
The content of TDCET

TDCET is a series of exercises based on the theory of PSSEs and consists of two parts (Fig. 3):1) integrated outpatient treatment, including manual fascia relaxation therapy, stretching, three-dimensional self-correction exercises based on the pathological mechanics of IS (including longitudinal stretching and pelvic adjustments in the coronal plane to correct the lateral curvature, physical exercises for the sagittal profile to improve thoracic spinal extension range of motion, and exercises with a wedge pad to modify the “humpback” posture, and horizontal trunk and pelvic rotations) and breathing training, and 2) family rehabilitation, which includes self-correcting exercises and basic body awareness therapy to learn the correct postures that need to be adopted in everyday life activities. The participants in the exercise group will perform 60-min integrated outpatient treatment 1–3 times a week under the guidance of a physical therapist in an outpatient clinic and 40-min family rehabilitation per day under the supervision of the parents at home. In addition, the parents will send the exercise records to the physiotherapist through the Micro Message Public Platform.

#### Generalized exercise

The participants in the CG will perform generalized exercise (GE) for a 60-min period 1–3 times a week under the guidance of a physical therapist in an outpatient clinic and 40-min self-correcting exercises per day under the supervision of the parents at home and the exercise records will also be sent to the physiotherapist through the Platform. CE will consist of a series of low-impact stretching and strengthening activities, such as curl-up, dead bug, side bridge, and birddog exercises [19].

#### Bracing

For all moderate IS patients, the Chêneau brace will be worn for not less than 22 h a day. The treatment regimen will last for 12 months.

### Incompliance or termination of the allocated intervention

Any subject who does not follow or complete the intervention period as specified in the protocol will be considered an incompliant subject.

The following are possible reasons for incompliance or termination:

1. Poor intervention compliance will be considered if the level of compliance to the treatments in the outpatient clinic is less than 80%. Compliance = (total number of outpatient treatments/minimum required number) × 100%.
2. Patients who consume drugs or participate in interventions that are not permitted during the clinical study, including other hormones (e.g., somatotropic hormone) and therapies (e.g., chiropractic and osteopathy) not mentioned in the trial, will not continue the study.
3. Patients with incomplete information due to a failure to complete the entire course of treatment for any reason will not continue the study.
4. Patients in whom scoliosis progresses at a rapid pace and surgery is recommended will not continue the study.
5. Patients with accidental injuries (such as sprains, fractures, and traumatic injuries) making it inappropriate to continue the trial and need to discontinue treatment will not continue the study.

The following measures should be taken:

1. An attempt to contact the subject should be made to ask for an explanation for incompliance or termination, and the results of the last assessment should be recorded.
2. For patients who need to withdraw from testing due to anaphylaxis, adverse reactions, or treatment failure, appropriate treatment measures should be taken immediately.
3. A form should be completed to explain the reason for study termination or incompliance.
4. Intention-to-treat (ITT) analyses will be performed for incompliant patients, as appropriate. If the incompliance rate exceeds 10%, the reason for incompliance will be explained in detail.

### Adverse events

Every adverse event (AE) that occurs during the trial will be faithfully recorded and evaluated to assess the associations between AEs and the experimental interventions or evaluations. Necessary measures will be taken immediately for the safety of the participates and to determine whether to stop the intervention. If any AEs occur during the study, the investigator will take necessary measures according to the patient’s condition in a timely manner.

### Outcome measures

The study will be conducted over a period of 12 months. All outcomes will be assessed at baseline and after the 12-month intervention. In total, one primary outcome indicator and four secondary outcome indicators will be analysed:

1. The primary outcome indicator is the change in the largest Cobb angle.

It is recommended that the magnitudes of the scoliotic curves are measured using the Cobb method. The Cobb angle will be measured on the standing frontal radiograph according to the Cobb method by a physician specializing in the treatment of scoliosis.

2) The secondary outcome measures will be as follows:
  - The sagittal index (SI) is the sum of the plumbline distances at C7 and L3. When SI<60 mm, the back is considered flat; 60–95 mm is in the normal range; and > 95 mm is considered kyphosis [20].
  - Forced vital capacity (FVC) is an established measure of pulmonary function.
  - Peak oxygen uptake (VO_2_peak) is recognized as the best expression of exercise endurance and will be measured by cardiopulmonary exercise testing.
  - Quality of life will be evaluated by the Scoliosis Research Society-22 (SRS-22) questionnaire. This questionnaire was specifically designed for patients with scoliosis and consists of 22 items addressing 5 dimensions: function/activity, pain, self-image, mental health, and satisfaction with treatment. The higher the score is, the better the outcome.

### Data management

All the involved participants were coded with a number. We used a case report form (CRF) to record the information of participants, including basic information,

X-ray results, functional evaluation results, and follow-up. The data will be collected by a blinded physiotherapist, other researchers will be forbidden from obtaining the research data. Besides, statisticians will be blinded in our study.

### Sample size calculation

The primary outcome indicator is the change in the largest Cobb angle. We calculated the sample sizes using the formula below:

$$\mathrm{n}1=\mathrm{n}2={\left[\left({\mathrm{Z}}_{2\alpha }+{\mathrm{Z}}_{2\beta}\right)\ast \mathrm{S}/\delta \right]}^2$$

1. Mild AIS: α = 0.05, Z_2α_ = 1.645;β = 0.10, Z_2β_ = 1.282. The previous clinical results showed that the combined standard deviation of the Cobb angle of the two groups was δ = 6.1°and S = 7.48 [21]. Based on these results, in this study, the sample size needed for each group was determined to be 26 cases if patients are included in the TDCET group and the CE group at a ratio of 1:1. Twenty percent more subjects will be required to allow adjustments for other factors, such as withdrawal, missing data, and loss to follow-up. It is recommended that a minimum of 33 subjects per group are included for mild IS comparisons.
2. Moderate AIS: α = 0.05, Z_2α_ = 1.645; β = 0.10, Z_2β_ = 1.282. The previous clinical results showed that the combined standard deviation of the Cobb angle of the two groups was δ = 2.82°and S = 3.75 [22]. Based on these results, in this study, the sample size needed for each group was calculated to be 31 cases if patients are included in the TDCET group and the CE group at a ratio of 1:1. Twenty percent more subjects will be required to allow adjustments for other factors, such as withdrawal, missing data, and loss to follow-up. It is recommended that a minimum of 39 subjects per group are included for moderate IS comparisons.

Hence, 66 patients with mild IS and 78 patients with moderate IS will be included.

### Statistical analysis

All statistical procedures will be performed according to the principles of intention-to-treat (ITT). Outcome assessor will be blinded after assignment to interventions. A linear mixed model will be used to identify the differences between the TDCET group and the CE group in the change of the largest Cobb angle, SI, FVC, VO_2_peak and SRS-22 questionnaire scores across the periods of assessment (intervention factors and time) in the mild and moderate subgroups. The Risser sign will be considered a covariate. When necessary, post hoc comparisons will be performed using the Bonferroni correction for multiple comparisons. The correlations between the clinical variables (Cobb angle) and secondary variables (SI, FVC, VO_2_peak and SRS-22 questionnaire scores) will be assessed. All data will be analysed using SPSS v.23.0 software. *P* < 0.05 will be considered statistically significant.

### Quality control and quality assurance

It is necessary to take appropriate measures to minimize bias because confounding bias is a key factor that can affect the quality of test results. Unified training and assessments will be conducted for participating researchers, and only strictly normative data will be used to reduce measurement bias.

In addition, the advantages and characteristics of the study will be explained to the subjects in detail before enrolment so that the participants and/or their parents will fully understand the possible benefits, thereby improving compliance and reducing withdrawal from the study.

It is necessary to use a three-grade quality control system: the researchers will conduct self-examinations of the cases they have included, the inspectors from each subgroup will supervise the assessments every month, and the inspectors from the research group will supervise the assessments every quarter.

A comprehensive Data and Safety Monitoring Board will be established to ensure the quality of each step during the testing process as much as possible. The Clinical Research Unit (CRU) is an independent committee responsible for controlling the quality and ensuring the safety of this study to ensure the safety of the subjects and the validity of the data. According to the outcomes, data quality, and evidence of AEs, the trials can be terminated if the intervention is considered useless or invalid. If AEs occur, the main investigator will decide whether to suspend the intervention in this study according to the patient’s condition. In addition, the DMC can terminate the trial if the data are found to be invalid or useless.

## Discussion

We adhered to the SPIRIT in the development and reporting of our protocol (Fig. 2). While PSSEs are routinely used in a number of central and southern European countries, most centres in other countries (mainly Anglo-Saxon countries) do not advocate their use. One of the reasons for this is that many health care professionals are not conversant with the differences between generalized physiotherapy exercises and PSSEs; while the former exercises are generic exercises usually consisting of low-impact stretching and strengthening activities such as yoga, Pilates and the Alexander technique, PSSEs consist of curve-specific exercises that are individually adapted to a patient’s curve site, curve magnitude and clinical characteristics [23]. Previous studies have reported that PSSEs positively affect the Cobb and trunk rotation angles, body symmetry, muscle balance and cosmetic trunk deformities in IS [24]. On the basis of the quality of the available studies, the final conclusion of the Cochrane review on the effect of exercises for scoliosis was that it is not possible to recommend the use of PSSEs for IS [13].

The TDCEs, which is an innovative therapy based on the theory of PSSEs and characterized by the combination of outpatient treatment and family rehabilitation (including basic body awareness therapy), was not sufficiently studied in the scientific literature, and the ideal conservative therapeutic schedules estimated for the therapy are controversial. A pervious study indicated that basic body awareness therapy may provide statistically significant improvements in thoracic, lumbar, and total curvatures (Cobb angles) [25], and Manticone et al. reported that task-oriented and active corrective exercises are more effective than traditional exercises in reducing spinal deformities (5^°^ reduction in the Cobb angle) and improving quality of life in mild IS patients [24]. This positive finding suggests that curve reduction may be due to increases in the strength and endurance of postural muscles and increased spine stability caused by activating postural muscles in active positions (anterior balance of the body) during exercises. Additionally, the exercises are based on selective movements designed to achieve the maximum possible correction of the deformity, and their postural effectiveness is strengthened by the development of neuromotor abilities during everyday activities. Therefore, TDCET includes self-correcting exercises and basic body awareness therapy may help patients adopt supportive attitudes and appropriate behaviours, thus enhancing their compliance with exercises and yielding better outcomes.

The results of this study will demonstrate whether TDCET can improve outcomes in IS. This study has the potential to influence clinical practice worldwide, especially in locations where TDCET is not routinely prescribed for IS.

## Supplementary Information

**Additional file 1.** SPIRIT 2013 checklist: recommended items to address in a clinical trial protocol and related documents.

**Additional file 2.** Ethical Approval Document.

## Data Availability

The datasets used and/or analysed during the current study are available from the corresponding author on reasonable request.

## Abbreviations

AE: Adverse event
AIS: Adolescent idiopathic scoliosis
CRU: Clinical Research Unit
CG: Control group
EG: Experimental group
FVC: Forced vital capacity
GE: Generalized exercise
IS: Idiopathic scoliosis
ITT: Intention-to-treat
non-RCT: Non-randomized controlled trial
VO_2_peak: Peak oxygen uptake
PSSEs: Physiotherapeutic scoliosis-specific exercises
RCTs: Randomized controlled trials
SI: Sagittal index
SOSORT: Scoliosis Orthopaedic and Rehabilitation Treatment
SRS: Scoliosis Research Society
SRS-22: Scoliosis Research Society-22
TDCEs: Three-dimensional corrective exercises
