# Supplementary material for: Three-dimensional corrective exercise therapy for idiopathic scoliosis: study protocol for a prospective non-randomized trial
Source: BMC Musculoskelet Disord. 2022 Feb 5;23:118. doi: 10.1186/s12891-022-05057-7 (PMC8818201; doi:10.1186/s12891-022-05057-7)
Supplement: Supplementary file 2 — Additional file 2. Ethical Approval Document. [file 12891_2022_5057_MOESM2_ESM.pdf]

上海交通大学医学院附属新华医院医学伦理委员会审查意见

Approval Letter Ethics Committee of Xinhua Hospital Affiliated to Shanghai Jiaotong University School of Medicine

批件号：XHEC-C-2021-009-2 会议编号/日期

Approval Number Meeting No./Date: M2021-002/2020-03-09

研究方案/编号 Protocol Title/Number	脊柱侧凸三维矫正运动疗法疗效前瞻性临床研究<br>Prospective clinical study on the efficacy of three-dimensional corrective exercise therapy for idiopathic scoliosis
审查文件 Reviewed documents	一、初审 1.临床研究伦理审查申请表；2.临床研究方案（第2版/2021.01.28）；3.知情同意书（8-16岁儿童2.0版，2021.01.28）；4.知情同意书（法定监护人 第2.0版，2021.01.28）；5.病历记录表；6受试者招募广告；7.主要研究者简历。二、复审 1.复审申请；2. 临床研究方案（第3版/2021.03.24）；3.4.知情同意书（法定监护人 第3.0版，2021.03.24）；4.修改清单列表<br>**Preliminary review**: 1.Application for clinical trial ethic review; 2.Clinical trial protocol (Version2.0/2021.01.28); 3.Informed consent form(For children between 8-16 years old,Version2.0/2021.01.28);4.Informed consent form(For legal guardians,Version2.0/2021.01.28); 5.Case Report Form；6.Recruitment advertisement; 7.Principal Investigator Biography<br>**Re-review**: 1.Application for re-review; 2.Clinical trial protocol (Version3.0/2021.03.24); 3.Informed consent form(For legal guardians,Version3.0/2021.03.24); 4.List of modified files
临床试验批件号 CFDA No.	NA
研究药物/器械名称 Study Product	NA
试验类别<br>**Phase of study**	☐I期临床试验 ☐II期临床试验 ☐III期临床试验 ☐IV期临床试验 ☐生物利用度试验 ☐上市药临床试验 ☐其他（请注明）_____ ☐器械临床验证 ☐器械上市后试验 ☐诊断试剂 ■临床研究<br>☐Phase I clinical trials ☐Phase II clinical trial ☐Phase III clinical trial ☐Phase IV clinical trial ☐Bioavailability test ☐Marketed drug clinical trials ☐Other (please specify)_______________ ☐Device clinical validation ☐Device post-marketing trials ☐Diagnostic reagents ■Clinical studies
审查方式 Type of review	■会议审查（初审） ■快速审查（复审） ☐紧急会议审查<br>■Review Meeting(Preliminary review) ■Expedited review(Re-review) ☐Emergency meeting review
研究单位/主要研究 Study site /PI	上海交通大学医学院附属新华医院 康复医学科/杜青<br>Department of Rehabilitation Medicine, Xinhua Hospital/Qing Du
申办单位/资助单位 Sponsor	上海申康医院发展中心/第二轮申康三年行动计划 SHDC2020CR3041B<br>Clinical Research Plan of SHDC/No. SHDC2020CR3041B）

审查意见 Recommendation

· 伦理委员会于 2021 年 3 月 9 日对项目进行了初审。本次会议应到委员 19 人，实到 19 人，回避 0 人。审查结果：必要修改后同意。审查意见：XHEC-C-2021-009-1.

· 2021 年 4 月 2 日收到复审申请及文件。审查结果：同意。请严格按照审查通过的试验方案和知情同意书开展本临床试验。

· 踪审查频率：□3 个月 □6 个月 ■12 个月 □不适用

· On March 9th, 2021, the Ethics Committee conducted a preliminary examination of the project. At this meeting, 19 members should be present, 19 actually, and 0 avoided. Examination results: Agreed after necessary modification. Review comment: XHEC-C-2021-009-1.

On April 2, 2021, the Committee received the application for re-review and revised documents. The result of review is: agreed. Please implement the approved research protocol strictly.

The frequency of tracking review: □3 months □6 months ■12 months □NA

主任委员（签名）

Director of Ethics Committee (signature): Jingsong Li

上海交通大学医学院附属新华医院医学伦理委员会

Xinhua Hospital Ethics Committee Affiliated to Shanghai Jiaotong University School of Medicine

2021 年 4 月 8 日 April 8, 2021

注意事项（请仔细阅读）Notes：

1. 已批准项目应遵循本伦理委员会批准的方案执行，应遵循国家药品监督管理局、国家卫生健康委《药物临床试验质量管理规范》(2020)、《医疗器械临床试验质量管理规范》(2016)、国家卫生计生委《涉及人的生物医学研究伦理审查办法》(2016)、WMA《赫尔辛基宣言》(2013) 和 CIOMS/WHO《涉及人的健康相关研究国际伦理准则》(2016) 和 ICH-GCP 等相关法律法规。The approved project shall be executed in accordance with the plan approved by the Ethics Committee. Should follow the national drug administration, the national health committee, the quality control standard for clinical trials (2020), the quality of medical instrument clinical trial management norms "(2016), the national health and family planning commission" relating to the people of biomedical research ethics review method "(2016), WMA the declaration of Helsinki (2013) and CIOMS / WHO International Code of Ethics on Research Related to Human Health (2016), ICH-GCP and other relevant laws and regulations.
2. 本临床试验应在伦理委员会批准之日起一年内实施，逾期未实施的，本批件自行废止。This clinical research should come into force within 1 year from the date of approval. Overtime will be automatically invalid.
3. 研究过程中，对研究方案和知情同意书等相关文件所做的任何修改，需提交伦理委员会审查。During the study, any changes made to the study protocol, informed consent and other relevant documents shall be submitted to the ethics committee for review.
4. 为了消除对受试者的紧急危害，在未获得伦理委员会同意的情况下，研究者修改或者偏离试验方案，

应当及时向伦理委员会、申办者报告，并说明理由，必要时报告药品监督管理部门。In order to eliminate the urgent harm to the subjects, if the researcher modifies or deviates from the test protocol without the approval of the ETHB, he/she should report to the ETHB and the sponsor in time, and explain the reasons, and report to the drug regulatory authority when necessary.

5. 研究者收到申办者提供的临床试验的相关安全性信息后应当及时签收阅读，并考虑受试者的治疗，是否进行相应调整，必要时尽早与受试者沟通，并应当向伦理委员会报告由申办方提供的可疑且非预期严重不良反应。After receiving the relevant safety information of the clinical trial provided by the sponsor, the investigator should sign and read it in a timely manner, consider the treatment of the subjects and whether to make corresponding adjustments, communicate with the subjects as soon as necessary, and report any suspicious and unexpected serious adverse reactions provided by the sponsor to the ethics committee.
6. 申办者终止或者暂停临床试验，研究者应当立即向临床试验机构、伦理委员会报告，并提供详细书面说明。If the sponsor terminates or suspends the clinical trial, the investigator shall immediately report to the clinical trial institution and the ethics committee, and provide detailed written explanations.
7. 伦理委员会终止或者暂停已经同意的临床试验，研究者应当立即向临床试验机构、申办者报告，并提供详细书面说明 If the IRC terminates or suspends the approved clinical trial, the investigator shall immediately report to the clinical trial institution and sponsor, and provide detailed written explanation
8. 研究者应当向伦理委员会提交临床试验的年度报告。The investigator shall submit an annual report of the clinical trial to the IRB.
9. 出现可能显著影响临床试验的实施或者增加受试者风险的情况，研究者应当尽快向申办者、伦理委员会和临床试验机构书面报告。临床试验完成后，研究者应当向伦理委员会提供临床试验结果的摘要。
10. The investigator should report in writing to the sponsor, the ethics committee, and the clinical trial agency as soon as possible any circumstance that could significantly affect the implementation of the clinical trial or increase the risk to subjects. Upon completion of the clinical trial, the investigator shall provide the IRB with a summary of the clinical trial results.

上海交通大学医学院附属新华医院 医学伦理委员会

地址：上海市杨浦区控江路 1665 号 邮编：200092 电话：021-25076143 传真：021-25078922
Address:No.1665 Kongjiang Road Shanghai, P.R China,Postcode:200092 Tel: +86-21-25076141 Fax: +86-21-25078922
